# German version of the Death Attitudes Profile- Revised (DAP-GR) – translation and validation of a multidimensional measurement of attitudes towards death

**DOI:** 10.1186/s40359-019-0336-6

**Published:** 2019-09-11

**Authors:** Jonas Jansen, Christian Schulz-Quach, Nikolett Eisenbeck, David F. Carreno, Andrea Schmitz, Rita Fountain, Matthias Franz, Ralf Schäfer, Paul T. P. Wong, Katharina Fetz

**Affiliations:** 1 Medical Faculty, Clinical Institute of Psychosomatic Medicine and Psychotherapy, University Hospital Düsseldorf, Düsseldorf, Germany; 2 Städtische Kliniken, Lukaskrankenhaus Neuss GmbH, Medical Clinic II, Neuss, Germany; 3 Department of Supportive Care, Princess Margaret Cancer Centre, University Health Network, Toronto, ON Canada; 4 Department of Psychiatry, University of Toronto, Toronto, ON Canada; 5 Department of Psychological Medicine, King’s College, Institute of Psychiatry, Psychology and Neuroscience, London, UK; 6 Karoli Gaspar University of the Reformed Church in Hungary, Budapest, Hungary; 7 Universidad de Almería, Almería, Spain; 8 LVR Clinic of Psychiatry, Psychosomatic and Psychotherapy for Children and Adolescence, Viersen, Germany; 9 Psychosocial Oncology and Palliative Care Department, Dana-Farber Cancer Institute, Boston, MA USA; 10 The Meaning Centered Counseling Institute, Toronto, Canada; 11 Chair of Research Methodology and Statistics, Department of Psychology and Psychotherapy, Faculty of Health, Witten/Herdecke University, Witten, Germany

**Keywords:** Death attitudes, Death anxiety, Death acceptance, Denial of death, Multidimensional measure, Death attitude profile-revised, Cultural adaption, DAP-GR, Factor analysis, Validation, Test construction

## Abstract

**Background:**

In Germany, only limited data are available on attitudes towards death. Existing measurements are complex and time consuming, and data on psychometric properties are limited. The Death Attitude Profile- Revised (DAP-R) captures attitudes towards dying and death. The measure consists of 32 items, which are assigned to 5 dimensions (Fear of Death, Death Avoidance, Neutral Acceptance, Approach Acceptance, Escape Acceptance). It has been translated and tested in several countries, but no German version exists to date. This study reports the translation of the Death Attitudes Profile-Revised (DAP-R) into German (DAP-GR) using a cross-cultural adaption process methodology and its psychometric assessment.

**Methods:**

The DAP-R was translated following guidelines for cultural adaption. A total of 216 medical students of the Heinrich Heine University Duesseldorf participated in this study. Interrater reliability was investigated by means of Kendall’s W concordance coefficient. The internal consistency of the DAP-GR Scales was assessed with Cronbach’s alpha coefficients. Split-half reliability was estimated using Spearman-Brown coefficients. Convergent validity was measured by Spearman’s correlation coefficient. Content validity was assessed by means of confirmatory factor analysis (CFA). All statistical analyses were performed using SPSS 24 and AMOS 22.

**Results:**

The items showed fair to good interrater reliability, with W-values ranging from .30 to .79. Internal consistency of the five subscales ranged from .61 (Neutral Acceptance) to .94 (Approach Acceptance). Split-half reliability was good, with a Spearman-Brown-coefficient of .83. The results of CFA slightly diverged from the original scale.

**Conclusion:**

Our results suggest overall good reliability of the German version of the DAP-R. The DAP-GR promises to be a robust instrument to establish normative data on death attitudes for use in German-speaking countries.

## Background

Examining people’s attitudes towards death and dying in Germany requires research not only to concentrate on optimizing medical care but also to address social, cultural, religious and ethnic circumstances [1]. Many people do not think about death much. However, when prompted to consider the idea of death, most people describe a feeling of apprehension or discomfort. Reactions range between anxiety, denial and acceptance of death [2, 3]. Hence, this study focuses on the different attitudes people express towards death. The public discourse project “30 thoughts on death” (http://www.30gedankenzumtod.de [German website]) is a joint research project between universities in Germany and follows the call for research and public dialogue on this topic [4].

It is often during the diagnosis of a life-limiting disease that people consciously ponder thoughts of personal dying and death for the first time [5]. Once people are confronted with death, primary anxious affect seems to be a natural response to death awareness. Nyatanga and de Vocht [6] (p. 412) define death anxiety as “an unpleasant emotion of multidimensional concerns that is of an existential origin provoked on contemplation of death of self or others”. [5] describes the essential function of anxiety as reparative. While a low level of anxiety can be motivating, a high level can have detrimental effects. Prolonged overt anxiety can lead to a state of terror or existential dread. Following Terror-Management-Theory (TMT) research, the failure of protective psychogenic mechanisms and defence strategies that aim to bolster self-esteem and ultimately reduce the experience of anxiety leads to overt annihilation anxiety [7, 8]. In accordance with TMT, individuals who have high self-esteem and strong worldview beliefs often do not think about death much or fear it consciously. These individuals often express an attitude of death acceptance. However, Wong and Tomer (1999) argued that a meaning-oriented approach towards death acceptance may reduce the terror of death. In this context, [9, 10] presented his meaning-management theory (MMT) of death acceptance. MMT is rooted in existential-humanistic theory [11] and constructivist perspectives [12], but it also incorporates cognitive-behavioural processes. It is a comprehensive psychological theory about how to manage various meaning-related processes to meet basic needs for survival and happiness.

Wong et al. [13] developed the Death Attitude Profile-Revised and identified three types of death acceptance: Neutral Acceptance (accepting death as a natural process of life), Approach Acceptance (looking forward to a blessed afterlife) and Escape Acceptance (accepting death as a better alternative to present sufferings). Research has shown that Neutral or Approach death acceptance correlates with personal meaning; that is, individuals who see their lives as fulfilling have consistently been found to express less death anxiety [13–21]. One relevant application of the DAP-R measure lies in its ability to measure these different attitudes to provide a more nuanced understanding of how individuals react in situations of death confrontation and mortality salience, such as when they are confronted with a diagnosis of a life-limiting illness or when working around death and dying is part of their professional role description, such as in hospice and palliative care [22].

In Germany, only limited data are available on attitudes towards death, and existing measurements are not easily applicable. The existing measurements are complex and time consuming, and data on psychometric properties are limited [23–25]. The DAP-R has been translated and tested in several countries, but no German version exists to date. Hence, in this study, we report the translation and adaption of the previously validated DAP-R measure into German using a cross-cultural adaption process methodology [26].

In this study, the researchers focus on medical students since Undergraduate Palliative Care Education (UPCE) has become mandatory in Germany in recent years. Furthermore, medical students are particularly interesting since they are in a unique transition state between being part of the general public and becoming medical professionals [27]. Another study by our research group found that students wish to have death education as part of end-of-life care (EOLC) [28]. We believe that the DAP-GR could foster the opportunity to realize that wish in German-speaking countries.

The researchers opted against using a palliative care sample since it might have been difficult to recruit a comparable sample of patients in the same time frame. The objectives of this study were on the one hand to report the translation of the Death Attitudes Profile-Revised (DAP-R) into German (DAP-GR) using a cross-cultural adaption process methodology and on the other hand to evaluate the psychometric properties of the German adaptation of the DAP-R in a sample of medical students. We analysed the face validity, confirmatory factor structure, the replicability of the dimensions and the internal consistency. In a first part of the study, a small sample of medical students helped to empirically determine the face validity of the proposed five dimensions of the DAP-GR. In the second part of the study the main sample, with over 200 participants, were used to analyse the confirmatory factor structure, the replicability of the dimensions and the internal consistency.

## Methods

### Sample

More than 200 medical students of the Heinrich Heine University Duesseldorf who were at least 18 years of age or older and sufficiently fluent in the German language participated in this study. The demographic data of the face validity sample (*n* = 32) and the 216 participants of the main sample are presented in Table 1. In the face validity sample, the majority of the students were female (65,6%). Their average age was 27,41 years (SD = 3,69). For this part of the study, we included only students from higher semesters (> 5 semesters), of whom 78,1% reported having a fundamental spiritual belief.

**Table 1:** Sample characteristics for face validity and main sample

Variables	*Face validity (N = 32)* *M (SD) [range] / %*	*Main sample (N = 216)* *M (SD) [range] / %*
Age	27.41 (3.69) [22–27]	24.37 (3.92) [18–39]
Gender		
Female	65.6	63.0
Male	34.4	37.0
Semester		
1	–	–
2	–	13.4
3	–	2.3
4	–	24.5
5	–	3.3
6	3.1	6.0
7	6.3	2.3
8	6.3	.6
9	28.1	12.5
10	34.4	20.8
11	6.6	.9
12	9.4	3.2
> 12	6.3	3.7
Spiritual beliefs (%)		
Roman Catholic	59.4	32.9
Protestant	9.4	23,3
Christian orthodox	–	2.9
Muslim	3.1	3.8
Buddhist	6.2	1.9
Jehovah’s Witnesses	–	.5
Atheist	21.9	11.9
Non	–	22.9
Experience with Dying/ Death	96.9	84.7
Personally involved in topics Dying/Death in the last four weeks	21.9	17.6

Note: Percentages of spiritual beliefs of main Sample based on N 210, since missing responses

For the main sample, most of the participants were female (63%), and the average age was 24.37 years (*SD* = 3.92). We included participants from all semesters (see Table 1). A total of 66,2% reported having a fundamental spiritual belief. The majority had previous experience with dying or death but had not been personally involved in these topics in the last 4 weeks (see Table 1).

### Death attitude profile- revised

DAP-R [13] captures attitudes towards dying and death. The measure consists of 32 items, which are assigned to 5 dimensions. The measure is answered on a 7-point Likert scale (from 1 = strongly disagree to 7 = strongly agree), with each item beginning with either strongly disagree or strongly agree (random polarity pattern) to reduce possible acquiescence bias [29]. Total scores on each subscale are the average of the items of the subscale. The five dimensions are as follows.

1. *Fear of Death (Todesfurcht).* This dimension captures the fear of dying and death. Issues related to dying and death are complex and result from different reasons (e.g., “The prospect of my own death arouses anxiety in me”). The internal consistency of the original dimension was α = 0.86 (seven items: 1, 2, 7, 18, 20, 21 and 32).
2. *Death Avoidance (Vermeidungshaltung).* This dimension measures the avoidance of thoughts and feelings towards dying and death. It is important not to see death avoidance as the absence of the fear of death (e.g., “I always try not to think about death”). The internal consistency of the original dimension was α = 0.88 (five items: 3, 10, 12, 19 and 26)
3. *Neutral Acceptance (Neutrale Akzeptanz).* This dimension captures a neutral attitude towards dying and death. In this case, death is considered as an integral part of life (e.g., “Death should be viewed as a natural, undeniable, and unavoidable event”). The internal consistency of the original dimension was α = 0.65 (five items: 6, 14, 17, 24 and 30)
4. *Approach Acceptance (Akzeptanz von Tod als Schwelle zum Jenseits).* This dimension implies a belief in a happy afterlife (e.g., “I believe that I will be in heaven after I die”). The internal consistency of the original dimension was α = 0.97 (ten items: 4, 8, 13, 15, 16, 22, 25, 27, 28 and 31).
5. *Escape Acceptance (Akzeptanz von Tod als Ausweg).* This dimension captures positive attitudes towards death in light of suffering. When life is full of pain and distress, death may occur as a welcome alternative (e.g., “Death will bring an end to all my troubles”). The internal consistency of the original dimension was α = 0.84 (five items: 5, 9, 11, 23 and 29).

### Translation of the DAP-R

**Fig. 1:**
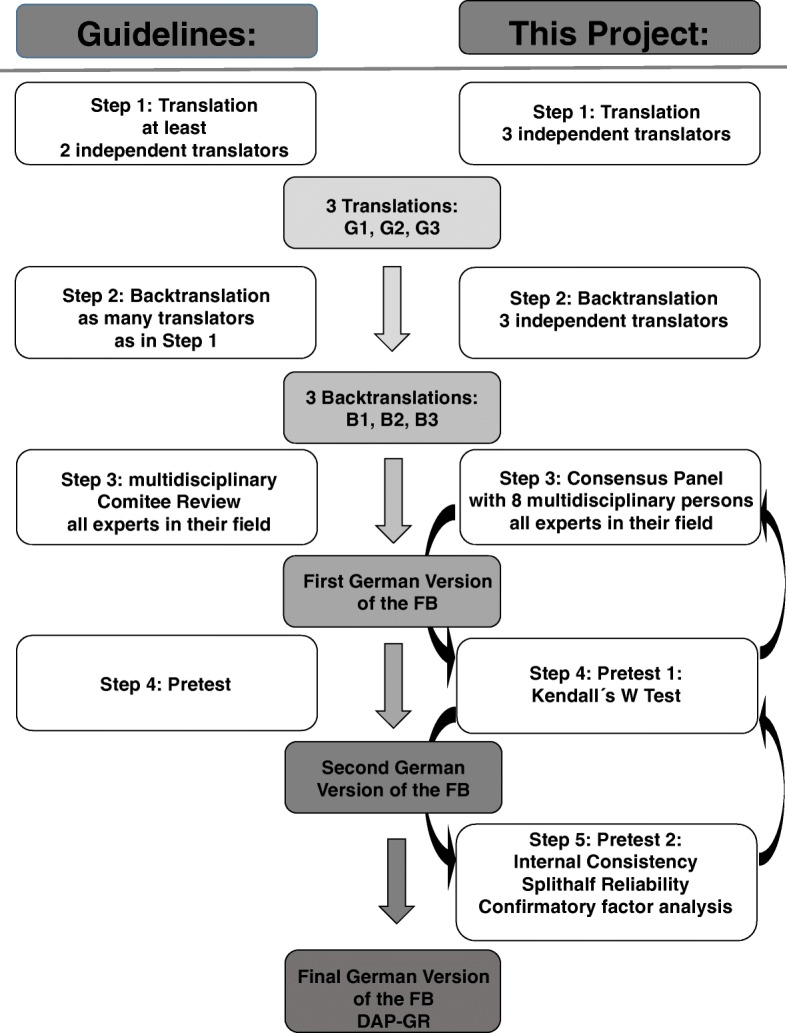
Flowchart Process adapted to: Guidelines for cultural adaption (Guillemin, 1993)

The DAP-R was translated following the proposed guidelines for cultural adaption by Guillemin et al. [26]. An overview of the translation process is shown in Fig. 1 (flowchart translation process). To study the health care needs of people with diverse cultural backgrounds, research instruments must be reliable and valid in each culture studied [30, 31]. If quantitative measures are used in research, it is necessary to translate these measures into the language of the culture being studied. Without verification of the adequacy of translation, differences found while using the target language version in the target population might be due to errors in translation rather than representing true differences between countries [32]. The original “Death Attitude Profile-Revised: A multidimensional measure of attitudes towards death” measure [13] was translated from English to German by three independent professional translators (target language versions (German): G1, G2, G3). According to [26], differing interpretations and translation errors of ambiguous items in the original can be detected by this procedure. If the translator is aware of the objectives underlying the measure, a more reliable restitution of the intended measurement can result, whereas translators who are unaware of these objectives may draw unexpected meanings from the original tool [33]. We used only qualified translators who translated into German, their mother tongue [34].

In a second step, the resulting German target versions G1-G3 of the measure were back-translated into English, again by three different independent professional translators, to reveal mistakes in the translation and to verify the semantic equivalence between the source language (SL) version and the target language (TL) version (back-translation versions B1, B2, B3). In the next step, we conducted a multidisciplinary consensus panel. The aim of this panel was to produce a preliminary final version of the German DAP-R (FB) that would be equal in semantic, idiomatic, empirical and conceptual ways based on the diverse forward- and backward translations described previously. Every participant in the panel received the original version of the DAP-R, the forward-translations G1-G3, the back-translations B1-B3, a proposed version by the head of the panel/research project, and guidelines on how to conduct the panel. The panel consisted of 9 participants, all of whom were experts in their field. Table 2 shows an overview of the panel participants and their expertise. The panel met on the 28th of March and the 9th of April in 2014, and a preliminary final version was produced on the 9th of April.

**Table 2:** Participants of the consensus panel and their expertise

Participants of the consensus panel	
Christian Schulz-Quach	Head of research project, Head of Panel, Medical expert for Palliative Care and Palliative Care Education
Jonas Jansen	Doctoral candidate, responsible for research project
Andrea Schmitz	Medical expert for Palliative Care and Palliative Care Education
Manuela Respondek	Nursing Expert for Palliative Care
Ursula Wenzel-Meyburg	Expert for Palliative Care Education
Alexandra Scherg	Student Expert for Palliative Care Education
Rita Fountain	Expert for Translation process
Collin MacKenzie	English Native speaker with teaching assignment at the University Hospital of Duesseldorf
Ralf Schäfer	Expert in Psychology (External Consultant)

### Procedure

To empirically determine the face validity of the proposed five dimensions of the DAP-R, we asked an independent group of 32 medical students of the Heinrich Heine University to place each item into what they believed was the most conceptually appropriate category. This part of the study was conducted via a paper/pencil method.

The main study took place at the Heinrich Heine University. Participants were asked to answer the measure using iPads. This survey mostly took place in the foyer of the medical special library of the Heinrich Heine University. Attendees provided informed consent for participation by finally transferring their results to our database via a button at the end of the survey.

### Data analysis

Face validity was investigated by means of Kendall’s W concordance coefficient test of interrater reliability [35].

For the main sample, prior to data collection, a power analysis concerning sample size for split-half reliability (bivariate correlation, two tailed) was performed by means of G-power [36], resulting in a suggested sample size of *N* = 138. For the confirmatory factor analysis, we set a sample size above 200 participants [37].

First, missing data on the DAP-R were evaluated. The amount of missing data was less than 1% in the case of each variable and was classified as being “missing completely at random” as Little’s Missing Completely at Random Test was not significant (χ2 (705) = 685.66, *p* = .692). Missing data were replaced with the expectation-maximization algorithm for each subscale.

After conducting descriptive statistics (means, standard deviations and ranges), the normal distribution of each subscale was evaluated with the Shapiro-Wilk test. The internal consistency of the DAP-R scales was assessed with Cronbach’s alpha coefficients. Split-half reliability was estimated using the Spearman-Brown coefficient. Correlations between the subscales were measured with Spearman’s correlation coefficient as the data were not normally distributed. Then, subsamples were assessed for systematic differences concerning age, gender, educational status (semester), educational background and prior experience with death.

Prior to confirmatory factor analysis, the data were checked for multivariate normality by means of analyses of kurtosis and skewness. In our sample, kurtosis and skewness data were close to zero and not close to 2 and 7 in any cases; thus, we assumed multivariate normality, except for one case (which was approximately skewness 5). The data typically were between − 1 and 1. In their classic article, Curran, West and Finch [38] defined moderate non-normality as skewness 2 and kurtosis 7. Moreover, because of the sensitivity of chi-square to non-normality and because it overestimates the lack of fit (type 1 error) when conducting CFA [39, 40], we report other descriptive fit statistics, such as TLI and CFI.

To conduct the confirmatory factor analysis, the covariance matrix was introduced to AMOS 22 [41]. After introducing the data, maximum likelihood estimation was used, and various goodness-of-fit estimations were analysed to assess the fit of the data: chi-square (χ^2^), χ2/degree of freedom ratio (CMIN/DF), Comparative Fit Index (CFI), Root Mean Square Error of Approximation (RMSEA) and Standardized Mean Square Residual (SRMR). As the χ2 statistic is sensitive to sample size issues overestimating the lack of fit, it was not relied upon as a basis for acceptance or rejection of the model (e.g., [39, 40]). Thus, the CMIN/DF is preferred instead, with values between 1 and 3 indicate a good-fitting model [42]. According to Hu and Bentler (1998), RMSEA values below .06 indicate a good fit, while other authors accept values below .08 as a reasonable fit of the model [43]. SRMR values below .08 are considered a good fit [44], while CFI values above .90 indicate an acceptable fit and those above .95 indicate an excellent fit of the model [42, 44, 45]. For the factor loadings, [37] suggested the following cut-offs: .32 (poor), .45 (fair), .55 (good), .63 (very good) and .71 (excellent).

## Results

### Face validity sample

The face validity results are shown in Table 3. Kendall’s W test revealed fair to good values, indicating acceptable inter-rater agreement and thus acceptable face validity.

**Table 3:** Results of Kendall’s W face validity

Item	Original item	German Translation	Kendall’s W	χ2
1	Death is no doubt a grim experience.	Der Tod ist zweifellos eine grauenvolle Erfahrung.	.42	53.19
2	The prospect of my own death arouses anxiety in me.	Die Aussicht auf meinen eigenen Tod verursacht mir Angst.	.30	37.22
3	I avoid death thoughts at all costs.	Ich vermeide Todesgedanken um jeden Preis.	.49	62.29
4	I believe that I will be in heaven after I die.	Ich glaube, dass ich nach meinem Tod in den Himmel komme.	.65	81.11
5	Death will bring an end to all my troubles.	Der Tod wird all meinen Sorgen ein Ende bereiten.	.59	75.97
6	Death should be viewed as a natural, undeniable, and unavoidable event.	Der Tod sollte als natürliches, unbestreitbares und unvermeidliches Ereignis angesehen werden.	.59	73.48
7	I am disturbed by the finality of death.	Die Endgültigkeit des Todes verstört mich.	.36	46.12
8	Death is an entrance to a place of ultimate satisfaction.	Der Tod stellt die Schwelle zu einem Ort der höchsten Zufriedenheit dar.	.67	85.92
9	Death provides an escape from this terrible world.	Der Tod bietet einen Ausweg aus dieser schrecklichen Welt.	.69	88.27
10	Whenever the thought of death enters my mind, I try to push it away.	Wann immer mir der Gedanke an den Tod in den Sinn kommt, versuche ich ihn beiseite zu schieben.	.63	80.13
11	Death is deliverance from suffering and pain.	Der Tod stellt die Erlösung von Schmerz und Leid dar.	.79	100.57
12	I always try not to think of death.	Ich bemühe mich stets, nicht an den Tod zu denken.	.57	72.68
13	I believe that heaven will be a much better place than this world.	Ich glaube, dass der Himmel ein viel besserer Ort sein wird, als diese Welt.	.63	80.97
14	Death is a natural aspect of life.	Der Tod ist ein natürlicher Aspekt des Lebens.	.65	83.58
15	Death is a union with God and eternal bliss.	Der Tod ist eine Vereinigung mit Gott und ewige Glückseligkeit.	.71	91.23
16	Death brings a promise of a new and glorious life.	Der Tod bringt das Versprechen auf ein neues und herrliches Leben.	.66	84.73
17	I would neither fear death nor welcome it.	Ich würde den Tod weder fürchten noch willkommen heißen.	.68	86.55
18	I have an intense fear of death.	Ich habe große Angst vor dem Tod.	.59	75.86
19	I avoid thinking about death altogether.	Über den Tod nachzudenken, vermeide ich komplett.	.65	83.24
20	The subject of life after death troubles me greatly.	Das Thema Leben nach dem Tod beunruhigt mich sehr.	.34	43.94
21	The fact that death will mean the end of everything as I know it frightens me.	Die Tatsache, dass der Tod das Ende von allem, wie ich es kenne, bedeuten wird macht mir Angst.	.35	45.03
22	I look forward to a reunion with my loved ones after I die.	Ich freue mich auf ein Wiedersehen mit mir nahestehenden Menschen, nachdem ich gestorben bin.	.71	90.51
23	I view death as a relief from earthly suffering.	Ich sehe den Tod als Erlösung von irdischem Leiden.	.72	91.95
24	Death is simply a part of the process of life.	Der Tod ist einfach ein Teil des Lebensprozesses.	.60	76.26
25	I see death as a passage to an eternal and blessed place.	Ich sehe den Tod als einen Übergang zu einem ewigen und gesegneten Ort.	.68	87.51
26	I try to have nothing to do with the subject of death.	Ich versuche nichts mit dem Thema Tod zu tun zu haben.	.63	80.10
27	Death offers a wonderful release of the soul.	Der Tod bietet eine wunderbare Befreiung der Seele.	.77	97.88
28	One thing that gives me comfort in facing death is my belief in the afterlife.	Eine Sache die mir Trost gibt wenn ich dem Tod ins Auge sehe, ist mein Glaube an das Leben nach dem Tod.	.60	75.50
29	I see death as a relief from the burden of this life.	Ich sehe den Tod als Erlösung von der Last dieses Lebens.	.72	91.96
30	Death is neither good nor bad.	Der Tod ist weder gut noch schlecht.	.65	83.21
31	I look forward to life after death.	Ich freue mich auf das Leben nach dem Tod.	.58	73.85
32	The uncertainty of not knowing what happens after death worries me.	Die Ungewissheit, über das was nach dem Tod passiert, beunruhigt mich.	.34	43.16

Note: all *df* = 4, all *p* < .01

### Main sample

#### Scale characteristics and reliability

The means and standard deviations of the five factors were similar to the data obtained in the original study of [13] (see Table 4). Although in most cases there were no problematic levels of skewness and kurtosis, the scales did not show a normal distribution (in each case, Shapiro-Wilk tests were *p* < .05). The internal consistency of the five subscales was in line with the original measure [13] and ranged from a low of .61 (Neutral Acceptance) to a high of .94 (Approach Acceptance) (see Table 4). Split-half reliability analysis also yielded good results as the Spearman-Brown-coefficient was .83.

**Table 4:** Descriptive statistics and intercorrelations between the subscales of DAP-GR

	Fear of death	Death avoidance	Neutral acceptance	Approach acceptance	Escape acceptance
Fear of death					
Death avoidance	.38^***^				
Neutral acceptance	- .39^***^	- .21^**^			
Approach acceptance	−.07	.00	- .10		
Escape acceptance	−.02	- .02	−.02	.31^***^	
*M*	3.97	2.77	5.70	3.42	3.54
*SD*	1.22	.98	.73	1.43	1.18
Range	1.14–6.43	1–6	2–7	.98–6.6	1–7
Kurtosis	- .46	- .46	2.64	- .80	- .24
Skewness	- .22	.80	- .88	.17	.32
Cronbach’s alpha	.82	.79	.61	.94	.75

Note: *N* = 216; * *p* < .050; ** *p* < .001; *** *p* < .0005. All *p* values are two-tailed

Similar to the original version, our data indicated that the factors were quite independent. Only the Fear of Death factor correlated positively with Death Avoidance, and both of them were negatively associated with Neutral Acceptance (see Table 4). There were no statistically significant differences concerning age, gender, semester, educational background and prior experience with death in any of the DAP-R subscales, *p* > .05.

#### Confirmatory factor analysis

**Fig. 2:**
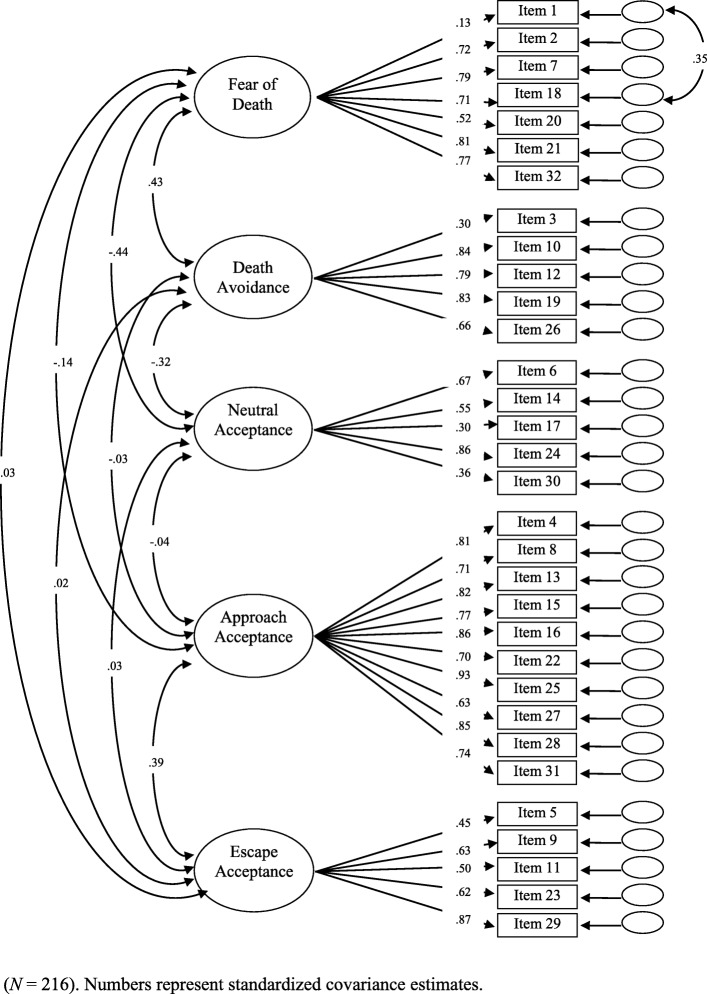
Five-factor confirmatory factor analysis model of the DAP-GR

The assumption about the five-factor structure of the instrument was assessed with confirmatory factor analysis on the data during the first assessment (T1, *n* = 216). The fit was on the border of being acceptable, *χ2* (454) = 811.74, *p* < .001, CMIN/DF = 1.79, CFI = .90, RMSEA = .06, SRMR = .08. Because of the possibly problematic fit, the standardized residual covariance matrix was assessed. The highest covariance was found between Items 1 and 18 (*MI* = 17.11). This connection makes sense between these two items as they have very similar meanings. Additionally, a number of medium-low covariances (*MI* between 10 and 15) were found in the factor of Approach Acceptance, showing that some of the items may be redundant in this factor. However, after allowing the error terms to correlate between Items 1 and 18, the model fit became good, *χ2* (453) = 791,461, *p* < .001, CMIN/DF = 1.74, CFI = .90, RMSEA = .05, SRMR = .08. The only acceptable indicator was the CFI, which is understandable as in the case of the DAP-R, some items and subscales do not correlate (see Table 4). Figure 2 depicts the standardized solution of the five-factor model with the addition of the correlation between the two error terms. The analysis of the factor loadings shown in Fig. 2 suggest that Item 1 with a factor loading of .13 (and possibly Item 3 with a factor loading as low as .30) may be removed from the model as it does not load on the factor “Fear of Death”. Further analysis showed that this item could not be placed on any of the remaining four factors. These data slightly diverge from the original scale as in that study, all items loaded at .40 or greater on at least one component [13].

## Discussion

This study reported the translation process of the German version of the Death Attitude Profile- Revised (DAP-GR), a multidimensional questionnaire to measure death attitudes, and its validation in German medical students.

With regard to the face validity, all items showed fair to good W values ranging from .30 to .79. The data of the main sample showed that the means and standard deviations were in line with the original study. Most of the participants were female, in accordance with statistical findings that show that in the year 2012, 65% of German university graduates in medicine were female [46].

In general, our data suggest overall good reliability of the German version of the DAP-R (DAP-GR). The subscales showed relatively high internal consistencies ranging from .65 to .88, and our data showed good split-half reliability of .83, which was not tested in the original version of the measure. Similar to the original version [13], the factors were quite independent; only the Fear of Death factor correlated positively with Death Avoidance, and both of them were negatively associated with Neutral Acceptance. Furthermore, the factors’ intercorrelations suggest that there might be a higher order factor structure present. Approach and escape acceptance seem to cluster together representing a dimension of positive aspects of death. A negative dimension seems to be composed by fear of death/death avoidance anchoring one end of this spectrum, and neutral acceptance anchoring the other. These overarching positive and negative attitudinal dimensions appear to be independent of each other. This implies that positive and negative attitudes towards death are not necessarily the direct opposites of one another. Similar patterns have been found in work on positive and negative emotions ins social psychology [47–50] and research on masculinity and femininity [51–53]. In future work the meaning and implications of this structure should be considered.

The scores of DAP-GR’s subscales did not differ based on age, gender, semester, educational background and prior experience with death. Thus, these variables seem to have no influence on attitudes towards dying and death. These data differ from the original study, in which [13] reported that older participants were less afraid and more accepting of death as a reality and as an escape than younger participants. In that study, females were also significantly more accepting of life after death and more accepting of death as an escape than males were. These findings may be surprising since other studies show that, for example, gender or prior experience with death have an influence on attitudes towards dying and death [27, 54]. For instance, woman have a more positive attitude towards death than men do [55]. This finding seems to be related to a general difference between men and women in their perceptions of health [56]. Regarding the factor “prior experience to death” it might be helpful to take a closer look on the special experience, a participant of the study had, to improve the predictive power of the participants’ answers. For example, a bad and negative experience might influence one’s attitude in another way than a good and positive one. For further studies, in which we will use the final instrument, we will incorporate that fact and will not only enquire if the participant had prior experience with death, but also find a way to assess the quality of the experience. It may also be surprising as other studies show that according to students’ opinions, death education plays an important role in Undergraduate Palliative Care Education (UPCE) to achieve a positive self-estimation of competence and self-efficacy [57–61].

In our German sample, the confirmatory factor analysis showed a good fit of the data to the original factor structure with minor adjustments allowing item covariations among Items 1 and 18 due to linguistic similarities. Although the fit was perfectly acceptable, Item 1 did not load highly on any of the factors; thus, our results may suggest the need to rethink the elimination of this item.

### Limitations

In addition to the significant results, there are some limitations that should be mentioned. The measurement only offers a quantitative approach to the field of attitudes towards death. For more in-depth results, qualitative studies (e.g., interviews, focus groups) could be more appropriate. Qualitative studies may not only help to deepen understanding of this field of study but also validate existing quantitative results [62, 63].

With regard to the aim of validating this measurement for use in palliative care settings, it should be noted that the investigation of the test’s goodness criteria has not been established with palliative care patients for two reasons. First, it was difficult to recruit a comparable sample of palliative care patients in the same time frame. Second, the researchers selected medical students since UPCE has become mandatory in Germany in recent years. Furthermore, medical students are particularly interesting since they are in a unique transition state between being part of the general public and becoming medical professionals [27].

Another limitation of this study is that the correlations meant to test convergent validity were not significant. This implies that more theoretical work may be needed to identify predictive relationships and to further examine the construct validity of this German version of the DAP-R (DAP-GR). Due to the very limited and complex existing measurements in the German language that might be related to attitudes towards death, the construct validity analysis was ruled out for the objectives of this study. Our research group is currently applying the German Version of the DAP-R (DAP-GR) via the discourse project website “30 Gedanken zum Tod”, funded by the Bundesministerium für Bildung und Forschung (BMBF). [64] To date (5/2018), more than 1200 individuals have participated online. This project is ongoing, and data from the survey will be reported separately in the future.

## Conclusion

In summary, the limitations and absence of existing measures to capture attitudes towards dying and death in the German language have led to the translation and adaption of the Death Attitude Profile-Revised (DAP-R) [13]. The German Version of the DAP-R (DAP-GR) promises to be a robust instrument to establish normative data on death attitudes for use in German-speaking countries.

## Data Availability

The datasets used and/or analysed during the current study are available from the corresponding author on reasonable request.

## Abbreviations

B1–3: Back-translation versions
BMBF: Bundesministerium für Bildung und Forschung
DAP-GR: German Version of the Death Attitude Profile-Revised
DAP-R: Death Attitude Profile-Revised
EOLC: End of life care
FB: Preliminary final version of DAP-GR
G1–3: Target language versions (German)
MMT: Meaning-Management Theory
SL: Source language version
TL: Target language version
TMT: Terror-Management Theory
UPCE: Undergraduate Palliative Care Education
